# VISTA Blockade Aggravates Bone Loss in Experimental Murine Apical Periodontitis

**DOI:** 10.3389/fimmu.2021.738586

**Published:** 2021-10-07

**Authors:** Fuhua Yang, Yifei Zhang, Zhi Chen, Lu Zhang

**Affiliations:** ^1^ The State Key Laboratory Breeding Base of Basic Science of Stomatology (Hubei-MOST) and Key Laboratory for Oral Biomedicine of Ministry of Education (KLOBM), School and Hospital of Stomatology, Wuhan University, Wuhan, China; ^2^ Department of Endodontics, School and Hospital of Stomatology, Wuhan University, Wuhan, China

**Keywords:** immune checkpoint, apical periodontitis, VISTA, bone loss, inflammation

## Abstract

V-domain Ig suppressor of T cell activation (VISTA) is a novel coinhibitory immune checkpoint molecule that maintains immune homeostasis. The present study explored the role of VISTA in human and murine inflammatory tissues of apical periodontitis (AP). VISTA was upregulated in inflammatory tissues of human AP. In mice, the expression of VISTA gradually increased with the development of mouse experimental apical periodontitis (MAP), the CD3^+^ T cells, CD11b^+^ myeloid cells, and FOXP3^+^ regulatory T cells also gradually accumulated. Moreover, a blockade of VISTA using a mouse *in vivo* anti-VISTA antibody aggravated periapical bone loss and enhanced the infiltration of immune cells in an experimental mouse periapical periodontitis model. The collective results suggest that VISTA serves as a negative regulator of the development and bone loss of apical periodontitis.

## Introduction

**Figure 1:**
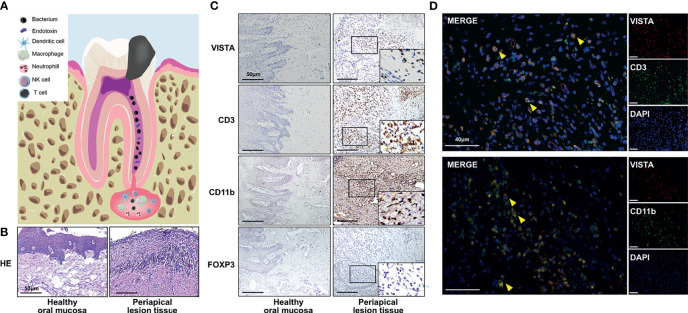
The expression of VISTA and immune cell infiltration in inflamed human periapical tissues. **(A)** Scheme of human apical periodontitis. **(B)** Hematoxylin-eosin staining of healthy human oral mucosa and periapical lesion tissue. **(C)** The accumulation of VISTA-, CD3-, CD11b-, and FOXP3-positive cells in healthy human oral mucosa (left panel) and periapical lesion tissue (right panel) (n=5). Scale bars=50 μm. **(D)** Colocalization staining of VISTA/CD3 and VISTA/CD11b in periapical lesions of human tissue. Scale bars=40 μm.

Apical periodontitis (AP) is characterized by sustained local inflammation around the root apex, which results in periapical alveolar bone loss (1). Exogenous bacteria activate the host immune system, leading to the recruitment of immune cells such as neutrophils, macrophages and T cells around the root apex (2). These immune cells secrete inflammatory chemokines and cytokines to modulate the inflammatory process (3). The infection is not completely obliterated by the host defense; thus, a sustained chronic inflammatory immune microenvironment involving bacterial virulence, immune cells, chemokines and cytokines is established (**Figure 1A**) (4).

Inflammation can disrupt physiological bone homeostasis around the root apex and result in inflammatory bone loss (2, 4). The balance between different subsets of immune cells could influence the outcome of infectious inflammatory osteolytic lesions (5). Activated T cells (such as Th1 and Th17) and other immune cells can highly express RANKL or release proinflammatory cytokines to promote osteoclastogenesis (6). Simultaneously, the innate immunoregulatory response against inflammation is mediated by other subsets of immune cells such as regulatory T cells (Tregs), which lead to the dampening of inflammatory and osteoclastogenic pathways (7). Many studies have successfully altered inflammatory bone loss by modulating the reaction of immune cells (8, 9).

As an important signaling pathway to curb the immune response and minimize inflammatory tissue damage, immune checkpoint molecules (ICs) expressed on immune cells actively participate in the maintenance of immune homeostasis (10). ICs play a central role in regulating cancer development, autoimmune diseases, etc. (11, 12). Moreover, recent reports suggest that ICs also participate in the regulation of inflammatory diseases (11). Our previous study demonstrated the overexpression of PD-1 and LAG-3 in periapical lesions, which suggests a potential role of ICs in inflammatory bone loss (13). In addition to the well-known ICs such as PD-1 and LAG-3, V-domain immunoglobulin suppressor of T cell activation (VISTA), which shows considerable homology with PD-1 and PD-L1, is another molecule that has recently attracted considerable interest (14). In contrast to PD-1 or LAG-3, which are mainly expressed on T cells, VISTA is more widely expressed on both myeloid cells and CD4^+^ and CD8^+^ T cells, acting not only as a receptor but also as a ligand. VISTA is involved in peripheral tolerance and mediates the function of immune cells in an unknown manner (15). A VISTA deficiency leads to a spontaneous proinflammatory phenotype in mice (16). The blockade of VISTA by specific *in vivo* antibodies augments the severity of autoimmune and inflammatory diseases such as asthma mouse models (17) and psoriasis mouse models (18). However, the *in vivo* function of ICs in a mouse experimental apical periodontitis model has not yet been elucidated, so we decided to investigate the role of VISTA in periapical diseases.

In the present study, we investigated the expression of VISTA in human AP tissue and a mouse experimental apical periodontitis (MAP) model. Moreover, VISTA was blocked using an *in vivo* anti-VISTA antibody in mice, and the alteration of inflammatory bone loss in the MAP model was investigated.

## Methods and Materials

### Ethics Statement

The experimental protocol using human tissue in the present study was approved by the Medical Ethics Committee of the School and Hospital of Stomatology at Wuhan University [2019 LUNSHENZI (A48)]. All the patients have signed the informed consent. The animal experimental protocol was approved by the Experimental Animal Ethics Committee of the School and Hospital of Stomatology at Wuhan University (S07920070L).

### Human Samples

Healthy oral mucosa and human inflammatory periapical tissues were collected from the Hospital of Stomatology of Wuhan University. Patients (n=5) who were diagnosed with periapical periodontitis were selected. The inclusion and exclusion criteria were as follows: a) The non-smoking patients (21-56 years) without systemic diseases, b) They had not been treated with antibiotics for at least 1 month before surgery, c) The patients had not received root canal treatment before, d) Obvious bone loss around the root apex was detected by Cone beam CT, e) The tooth was non-vital without spontaneous pain, f) The diagnosis was performed by experienced doctors based on the clinical symptoms of the patients. The patients underwent periapical surgery, and the inflammatory tissue around the root apex was removed and fixed with formalin for subsequent experiments. The healthy oral mucosa tissues (n=5) were obtained from residual mucosa after third molar extraction from non-smoking people which did not show any inflammation, as previously described (13, 19).

### Animal Experiments

Mouse experimental apical periodontitis was established using male C57BL/6 mice (6-8 weeks); After anesthesia, the first molar of the mice mandible was perforated using a 1/4 round bur until the mesial root canal could be detected by #6 K file, the pulp chamber was kept exposed to the oral cavity (20, 21). Mice were fed normally and euthanized at 7, 14, 21, and 28 days. The right side of the mandible was extracted for micro-CT scanning (Bruker SkyScan 1276), and the left side was formalin-fixed and decalcified in 10% EDTA for 4 weeks to prepare for the following experiments.

For the blockade experiment, mice were divided randomly into 8 groups (n=5): 4 experimental groups were treated with anti-VISTA antibody *in vivo*, and 4 control groups were treated with isotype control antibodies. At 7, 14, 21, and 28 days, an experimental group and a matched control group were euthanized; these time points are abbreviated as D7, D14, D21, and D28. *In vivo* anti-VISTA antibody (Bio X Cell, 13F3, BE0310) was intraperitoneally injected into mice twice a week (10 mg/kg), whereas isotype controls (Bio X Cell, N/A, BE0093) were used (10 mg/kg) as a control. Mice were monitored constantly to avoid physical and mental disorders

### Immunohistochemistry and Immunofluorescence

Formalin-fixed human tissues and decalcified mouse mandibles were dehydrated in an alcohol series and embedded in paraffin. The paraffin-embedded tissues were then cut into 4 μm slices. Heat-mediated antigen retrieval was performed following the manufacturer’s instructions. For immunohistochemistry (IHC), primary antibodies against CD3 (Cell Signaling Technology, CST 85061, Abcam, ab231775), CD11b (NB110-89474), FOXP3 (CST 98377, CST 12653), CXCL12 (Proteintech, 17402-1-AP) and VISTA (CST 54979) were used. A special kit for immunohistochemistry (Maxim KIT-9720) was used according to the manufacturer’s instructions. For immunofluorescence (IF), primary antibodies against CD3 (MAB4841), CD3 (17617-1-AP), CD11b (ab8878), and VISTA (CST 54979) were applied, and secondary antibodies against Cy3, 488, and 593 (Abbkine A23220, A22220, and Antgene ANT035s) were adopted.

### Micro-CT Scanning

Mouse right mandibles were extracted and desiccated for micro-CT scanning (Bruker SkyScan 1276). The scanning criteria were 55 kV and 200 μA with a 9 μm pixel size. The three-dimensional orientation of the samples was adjusted to ensure that each sample presented the same two-dimensional oriented section using a data viewer (Bruker). The volume of bone loss around the distal root apex of the first molar of each sample was calculated using CTan (Bruker).

### Western Blot Analysis

Mouse mandibles were collected and trimmed, only partially retained (as showed in **Figure 4A**, black doted rectangle) for the protein extraction (n=4), the procedures of Western blots were performed as described in our previous study (22). Primary antibodies used: anti-IL-6 (ab229381), anti-TNF-α (ABclonal, A0277).

### Blood Biochemistry

Mouse blood was obtained and left in static condition in room temperature for about 2 hours, faint yellow serum was separated after centrifugation (3000rpm, 15min), the blood biochemistry was performed (n=4 at D21, n=5 at D28) by servicebio (Wuhan, China): Aspartate transaminase (AST), alanine transaminase (ALT) for mouse liver function, blood urea nitrogen (BUN), creatinine (CR) for mouse renal function, the results were evaluated according to the reference range offered by servicebio (Wuhan, China).

### Statistical Analysis

All data are presented as the mean ± SEM. The data were analyzed and visualized using GraphPad Prism 5.0. The positive area and intensity of the immunostained slices were measured using ImageJ. Data analyses were conducted with the unpaired *t* test for two-group comparisons and one-way analysis of variance (ANOVA) for multiple group comparisons. P < 0.05 was defined as statistically significant.

## Results

### VISTA Was Expressed on T Cells and Myeloid Cells in Inflamed Human Periapical Tissues

To investigate the expression of VISTA in human AP, healthy human oral mucosa and inflamed periapical lesion tissues were collected (**Figure 1B**). Immunostaining indicated that the number of VISTA-positive cells sharply rose in inflamed periapical tissues compared with that of healthy oral mucosa (**Figure 1C**). We then detected different markers of immune cells. CD3 was strongly elevated in inflamed periapical lesions compared with that in healthy oral mucosa, which suggested infiltration of numerous T lymphocytes (**Figure 1C**). Additionally, CD11b was intensely expressed in inflamed periapical tissues, indicating the accumulation of many myeloid cells (**Figure 1C**). FOXP3, a putative marker of regulatory T cells (Tregs), was upregulated in AP, demonstrating that Tregs were also recruited to periapical lesions (**Figure 1C**).

To further illuminate the VISTA-positive cell subsets, double immunofluorescence was used. The localization of CD3 or CD11b partially overlapped with the localization of VISTA (**Figure 1D**), which indicated the accumulation of CD3^+^VISTA^+^ T cells and CD11b^+^VISTA^+^ myeloid cells in periapical lesions.

### The Expression of VISTA and Immune Cell Infiltration in Mouse Experimental Apical Periodontitis (MAP)

**Figure 2:**
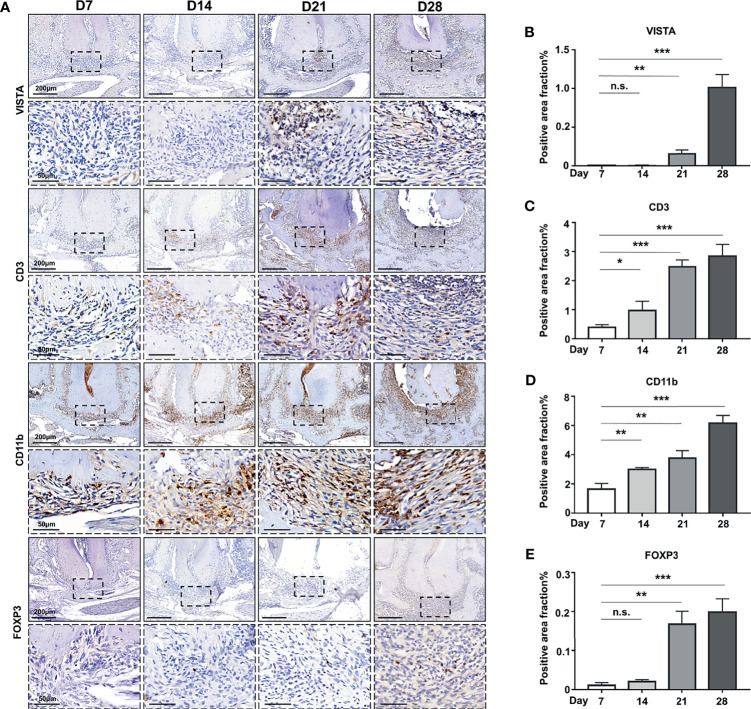
The expression of VISTA and immune cell infiltration in mouse experimental apical periodontitis. **(A)** The accumulation of CD3-, FOXP3-, CD11b-, and VISTA-positive cells at different time points in mouse experimental apical periodontitis tissues 7 days, 14 days, 21 days and 28 days after the mice underwent the pulp exposure procedure (n=5). Quantitative analysis of **(B)** VISTA-, **(C)** CD3-, **(D)** CD11b-, and **(E)** FOXP3-positive cells. Scale bars=200 μm or 50 μm at different magnifications, as shown in the images. n.s., not significant. *p < 0.05, **p < 0.01, ***p < 0.001.

**Figure 3:**
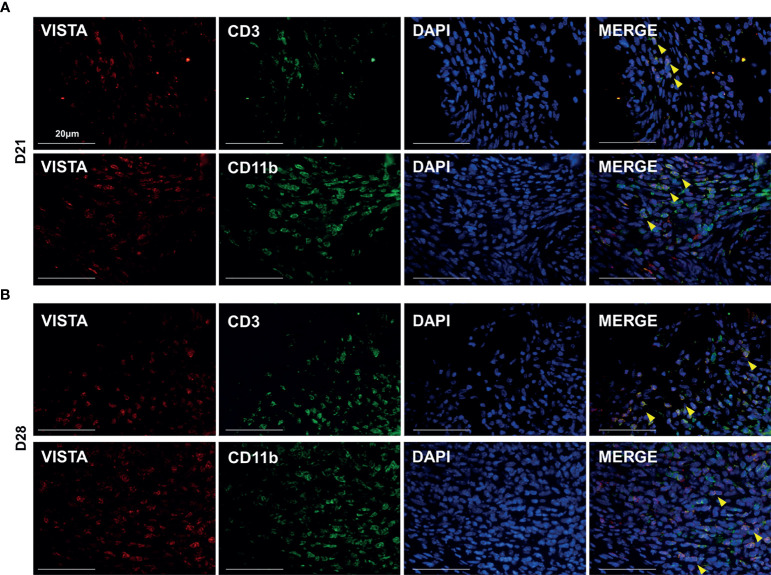
VISTA was expressed on both myeloid cells and T cells in mouse experimental apical periodontitis. Colocalization staining of VISTA/CD3 and VISTA/CD11b at different time points in experimental apical periodontitis tissues (n=5). **(A)** 21 days after the mice underwent the pulp exposure procedure and **(B)** 28 days after the procedure. Scale bars=20 μm.

To further investigate the potential function of VISTA in the development of AP, we established an experimental apical periodontitis mouse model to determine the expression pattern of VISTA and related immune cells. In the tissue from MAP, VISTA was barely detected at D7 and D14 (**Figure 2A**), but VISTA-positive cells were detected at D21 and further increased at D28 (**Figures 2A, B**). The expression of CD3 was gradually upregulated from D7 to D28 (**Figures 2A, C**), suggesting the gradual accumulation of T cells in the MAP. The expression pattern of CD11b was similar to that of CD3 (**Figures 2A, D**), indicating an increasing number of myeloid cells. The expression of FOXP3 resembled VISTA (**Figures 2A, E**), illustrating that Tregs were not recruited to the MAP until the lesions were present for a period of time. Double immunofluorescence was performed at D21 (**Figure 3A**) and D28 (**Figure 3B**). Consistent with human AP, the localization of CD3 or CD11b partially overlapped with the localization of VISTA, which indicated the accumulation of CD3^+^VISTA^+^ T cells and CD11b^+^VISTA^+^ myeloid cells in MAP.

### The Blockade of VISTA Exacerbated Osteolysis in Mouse Experimental Apical Periodontitis

**Figure 4:**
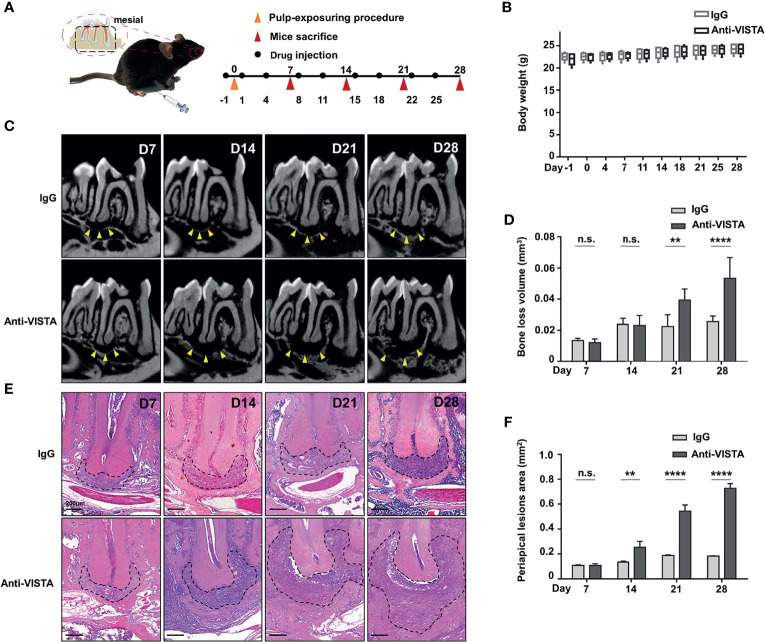
Blockade of VISTA exacerbated bone loss in mouse experimental apical periodontitis. **(A)** Schematic of experimental design (n=5). **(B)** Body weight of the anti-VISTA-treated group or IgG-treated group. **(C)** two-dimensional oriented sections of mouse mandibles at different time points of experimental apical periodontitis. **(D)** Bone loss volume around the distal root apex of mouse mandibular first molar at different time points. **(E)** Hematoxylin-eosin staining of apical tissue of mouse mandibular first molars. Scale bars=200 μm. **(F)** Quantitative analysis of periapical lesions area (indicated by black dash line) in **(E)** n.s., not significant. **p < 0.01, ****p < 0.0001.

To validate the role of VISTA in the bone loss of MAP, we adopted an antagonistic *in vivo* antibody against VISTA (anti-VISTA) to block the function of VISTA in MAP with isotype IgG as a control. A schematic shows the design of our blockade experiment (**Figure 4A**). Mouse body weights and physical and mental conditions were monitored regularly, and the body weights of the mice were not significantly influenced by the antagonist (**Figure 4B**). Additionally, no abnormality such as abnormal diet or inactivity was observed. The draining lymph nodes (cervical lymph nodes) and the spleens of anti-VISTA treated group were slightly swollen compared with control group (**Supplementary Figure 1E**). However, the blood biochemistry (n=4) showed that the liver function (ALT, AST) and renal function (BUN, CR) of the control group and treated group were not significantly influenced (**Supplementary Table 1.2**).

Micro-CT was applied to obtain both two-dimensional sections of mouse mandibles and statistics of three-dimensional bone loss volume. Two-dimensional sections of mice mandibles (**Figure 4C**) showed that the lesion area was not obviously changed at D7 but was slightly aggravated at D14 in the anti-VISTA group compared with that in the IgG group. The lesions were sharply exacerbated at D21 and D28 in the anti-VISTA group (indicated by yellow triangle). Hematoxylin-eosin staining of mandibular sections showed similar results (**Figures 4E, F**). However, the three-dimensional measurements (**Figure 4D**) showed that the change of bone erosion volume was not statistically significant at D7 and D14 (**Figure 4D**). Notably, there was significant bone erosion in the anti-VISTA group compared with that in the IgG group at D21 (P<0.01) and D28 (P<0.0001) in the three-dimensional bone loss volume analysis. These results suggest that anti-VISTA treatment significantly exacerbated bone loss in MAP at D21 and D28.

### The Blockade of VISTA Enhanced Immune Cell Infiltration in Mouse Experimental Apical Periodontitis

**Figure 5:**
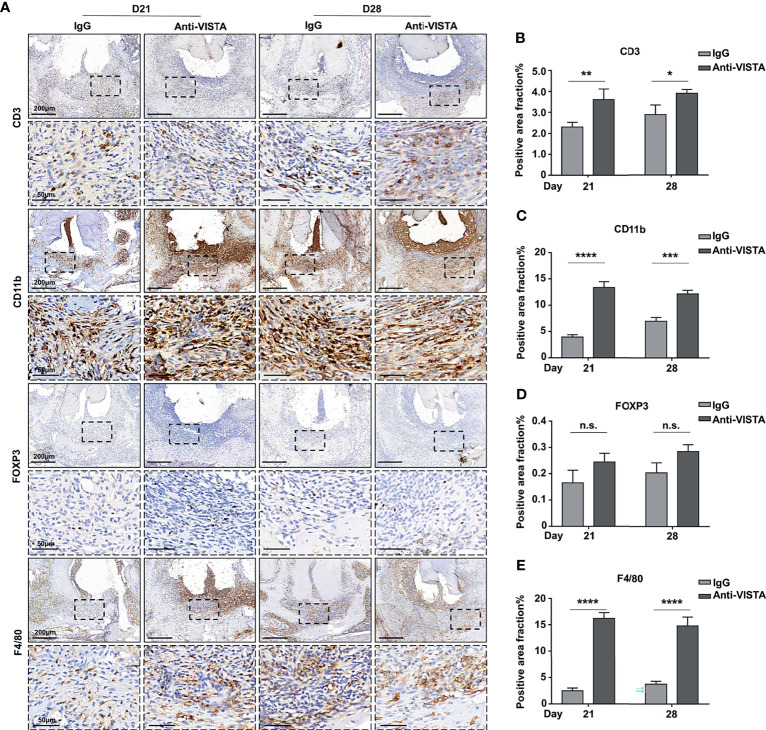
Blockade of VISTA enhanced immune cell infiltration in tissues of mouse experimental apical periodontitis. **(A)** The accumulation of CD3-, FOXP3-, CD11b-, and F4/80-positive cells in the anti-VISTA-treated or IgG-treated group at different time points in mouse experimental apical periodontitis tissues (n=5). Quantitative analysis of **(B)** CD3, **(C)** CD11b, **(D)** FOXP3, and **(E)** F4/80-positive cells. Scale bars =200 μm or 50 μm at different magnifications, as shown in the images. n.s., not significant. *p < 0.05, **p < 0.01, ***p < 0.001, ****p < 0.0001.

To further explore the mechanism of the exacerbated bone loss after anti-VISTA treatment, we investigated immune cell infiltration in both the anti-VISTA-treated groups (blockaded groups) and IgG-treated groups (control groups) at D21 and D28. The immunostaining of CD3^+^ T cells was increased in the blockaded groups compared with that of the control groups at both D21 and D28 (**Figures 5A, B**, P<0.01 at D21, P<0.05 at D28). The proportions of CD11b^+^ myeloid cells (**Figures 5A, C**; P<0.0001 at D21, P<0.001 at D28) and F4/80^+^ macrophages (**Figures 5A, E**; P<0.0001) were consistently significantly increased in the blockaded groups compared with those of the control groups at both D21 and D28. FOXP3^+^ Tregs were slightly increased in the VISTA-blockaded groups compared with those in the control groups at both D21 and D28; however, the increase of FOXP3^+^ Tregs did not reach statistical significance (**Figures 5A, D**). Furthermore, we detected the levels of cytokines and chemokines to evaluate the inflammatory status in both groups, IL-6 and TNF-α were upregulated in anti-VISTA-treated group compared with the control group at D28 (**Supplementary Figures 1A, B**). The level of CXCL12 was also elevated in anti-VISTA-treated group (**Supplementary Figures 1C, D**).

## Discussion

VISTA has been reported in some inflammatory and autoimmune diseases (23). In this study, we first demonstrated that VISTA is involved in the development of periapical periodontitis (AP). The present study shows that VISTA is expressed on CD3^+^ T cells and CD11b^+^ myeloid cells in human inflammatory periapical tissues. In mouse experimental apical periodontitis (MAP), VISTA-positive cells were not detected in the early stages (D7, D14) but gradually increased in the later stages (D21, D28). CD3^+^ VISTA^+^ T cells and CD11b^+^ VISTA^+^ myeloid cells were also detected in MAP. The specific *in vivo* blockade of VISTA aggregated bone erosion and immune cell accumulation around the mandibular apex at D21 and D28. These results suggest that VISTA plays a role in the development of AP and might act as a negative regulatory molecule.

The present study first demonstrated the participation of VISTA in exogenous bacteria-induced inflammatory bone disease. In the MAP model, the pulp chamber was perforated and left exposed to the mouse native oral microbial species, then the bone loss around the tooth apex was successfully induced over a period of time (20, 21, 24). As an important immune checkpoint protein, the role and function of VISTA has been mainly investigated in tumors and autoimmune or noninfectious inflammatory diseases (23). VISTA is highly expressed in tumor tissues (25–27), autoimmune disease lesions in systemic lupus erythematosus (18), ConA-induced mouse hepatitis (28), chemical agent-induced ear inflammation (29) and in the synovium of CAIA-induced arthritis (30). VISTA is mainly expressed on T cells and myeloid cells, but the expression patterns of VISTA vary depending on the disease.

Apical periodontitis is a type of inflammatory osteolytic disease (1). Inflammation is a defense mechanism of the host to kill and clear invasive pathogens. However, flood inflammation will cause uncontrolled bone tissue damage (5). To control overt immune responses thus maintaining immunological homeostasis and limiting damage by inflammation, essential intrinsic mechanisms of the host may inhibit excessive inflammation (31). Immune checkpoint molecules (ICs) are crucial for the maintenance of immune homeostasis in the host (12). VISTA expressed on myeloid antigen-presenting cells (APCs) combines with an unidentified receptor on CD4^+^ and CD8^+^ T cells to suppress their proliferation and cytokine production (15), and VISTA also suppresses the cytokine secretion of myeloid cells (32–34). VISTA expressed on CD4^+^ T cells limits T cell activation in a cell-intrinsic manner (25). In the present study, CD3^+^VISTA^+^ T cells and CD11b^+^VISTA^+^ myeloid cells were detected at D21 and D28 but not at D7 or D14 in MAP. The bone loss in MAP appeared to slow down in the later stage, the bone loss volume increased slowly from D21 to D28, which was consistent with previously reported results (35). These collective results suggest that the host invokes the expression of VISTA possibly as an intrinsic negative regulator to avoid excessive tissue damage during the development of MAP.

Regulatory T cells (Tregs) are innate, basic and crucial cells that suppress inflammation and regulate the immune response (36). Tregs have been previously reported to participate in the development of AP (37) and MAP (38). Consistent with these reports, we found that Tregs were expressed in AP. In MAP, we barely detected Tregs in MAP at D7 and D14. The number of Tregs was increased at D21 and D28, and the expression pattern was similar to that of VISTA. Tregs might coordinate VISTA-positive T cells and myeloid cells to restrain the development of MAP.

It has been indicated that the development of inflammatory bone loss can be mediated by modulating the intrinsic suppressing system (39). Bone loss in MAP was alleviated through the adoptive transfer of Tregs (8). In contrast, the blockade of Tregs induced heavier bone loss in experimental periodontitis (40). However, it is not clear whether the blockade of ICs could influence MAP. Antagonistic targeting of VISTA has been proven to be a unique activated avenue for immune cells (41). The blockade of VISTA may exacerbate the extent of inflammatory diseases by augmenting the proinflammatory role of T cells and myeloid cells (17, 18, 32, 42). Therefore, it is interesting to investigate whether anti-VISTA treatment affects the progression of MAP. The results showed that the blockade of VISTA worsened the extent of MAP at D21 and D28. It has been reported that the blockade of VISTA results in the accumulation of inflammatory cells in diseases targeting tissues (16, 29, 34). The present study also confirmed that the blockade of VISTA enhanced the recruitment of CD3^+^ T cells and CD11b^+^ myeloid cells (including F4/80^+^ macrophages). VISTA seems to be a pleiotropic cell checkpoint in the regulation of cytokines and chemokines (41), the deficiency of VISTA would result in the accumulation of cytokines and chemokines in mice (16, 18). In the present study, the elevated CXCL12 in anti-VISTA- treated group might contributed to the recruitment of immune cells at D28 in MAP. We also found IL-6 and TNF-α were upregulated in anti-VISTA group compared with the control group, which might indicate the more active inflammatory process in the treated group. The number of FOXP3^+^ Tregs increased slightly (not statistically significant), which might represent feedback from the exacerbated inflammatory microenvironment of MAP. Until now the cellular and molecular mechanisms of VISTA-mediated immune regulation are still not clear. Recent studies have presented several possible candidates such as VSIG3 (43). PSGL-1 (44) in human samples. However, more precise pathway of the regulative function of VISTA is still under investigation.

The present study revealed the potential negative regulatory mechanism of VISTA on inflammatory bone loss. The potential over-activation of VISTA of current anti-inflammatory drug may be underestimated and warrant explored. Furthermore, the VISTA-Ig has been applied on oxazolone-induced allergic mice dermatitis model and ameliorated the symptoms (45). Also, agonists of VISTA can reduce T cell response, prevent development of GVHD (46) and experimental asthma in mice model (17). These results provided a potential therapeutic implication by over-activating VISTA for therapeutic of inflammatory bone loss, which warrant further exploration in the future.

In conclusion, our study first demonstrated that VISTA might play a negative role in the development of AP, and that the development of AP might be affected by regulating VISTA-positive cells. Our study provides an increased understanding of AP and may inspire additional therapies for AP.

## Data Availability Statement

The raw data supporting the conclusions of this article will be made available by the authors, without undue reservation.

## Ethics Statement

The studies involving human participants were reviewed and approved by the Medical Ethics Committee of the School and Hospital of Stomatology at Wuhan University. The patients/participants provided their written informed consent to participate in this study. The animal study was reviewed and approved by the Experimental Animal Ethics Committee of the School and Hospital of Stomatology at Wuhan University.

## Author Contributions

FY contributed to the conception and design, data collection, data analysis and drafting the manuscript. YZ helped to collect data. ZC contributed to the conception and design. LZ reviewed the manuscript and managed the project. All authors contributed to the article and approved the submitted version.

## Funding

This work was funded by grants from National Natural Science Foundation of China (81974148, 81771064) to LZ and (81420108011) to ZC.

## Conflict of Interest

The authors declare that the research was conducted in the absence of any commercial or financial relationships that could be construed as a potential conflict of interest.

## Publisher’s Note

All claims expressed in this article are solely those of the authors and do not necessarily represent those of their affiliated organizations, or those of the publisher, the editors and the reviewers. Any product that may be evaluated in this article, or claim that may be made by its manufacturer, is not guaranteed or endorsed by the publisher.
