# Supplementary material for: VISTA Blockade Aggravates Bone Loss in Experimental Murine Apical Periodontitis
Source: Front Immunol. 2021 Oct 7;12:738586. doi: 10.3389/fimmu.2021.738586 (PMC8529274; doi:10.3389/fimmu.2021.738586)
Supplement: Supplementary file 1 [file DataSheet_1.docx]

Supplementary Material

## Supplementary Figures and Tables


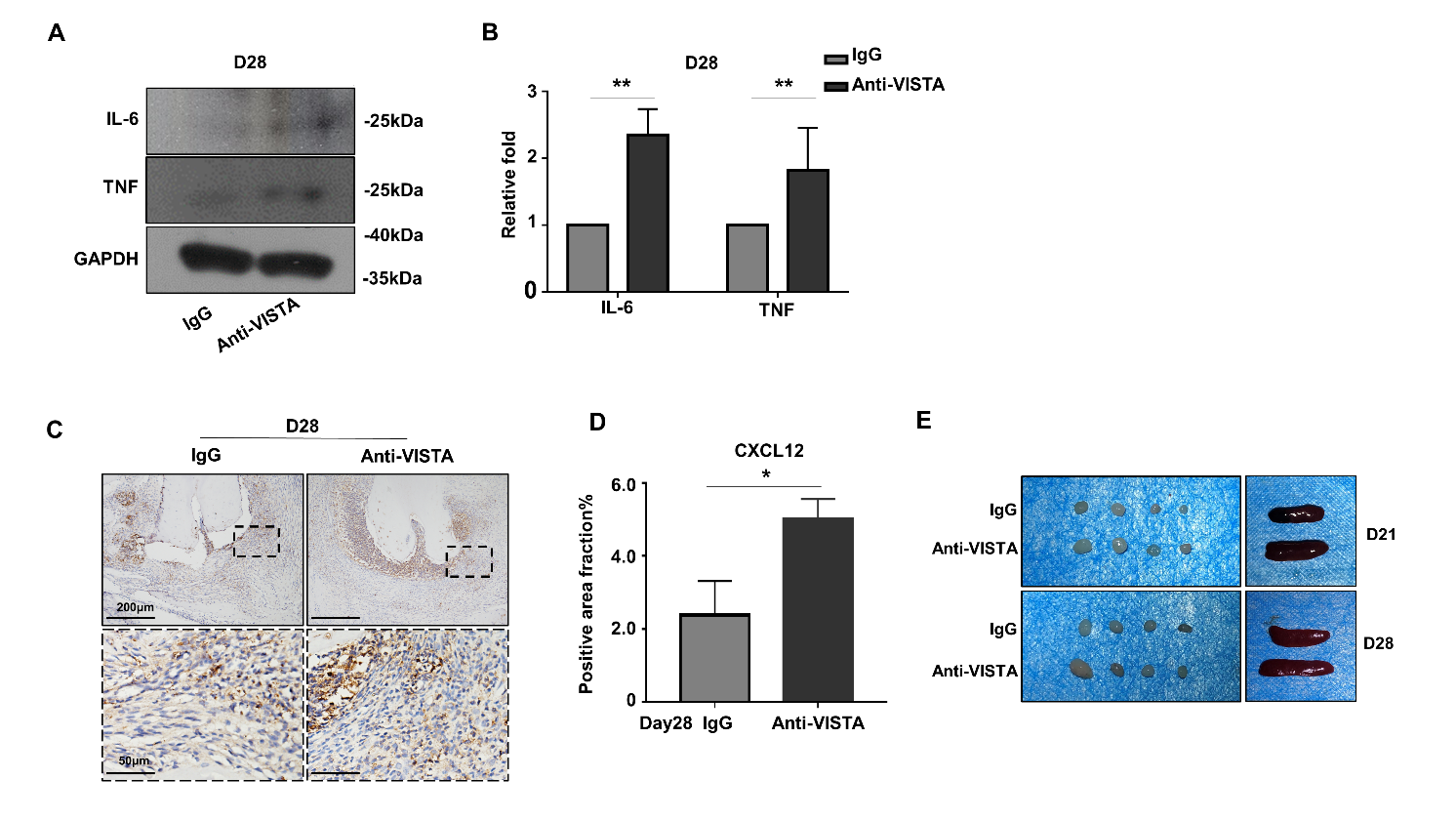


**Supplementary Figure 1.** (A). The expression of IL-6 and TNF-α in the mice mandibles (n=4) at D28. (B). Quantitative analysis of (A). **p < 0.01. (C). The immunohistochemistry staining of CXCL12 in mouse experimental apical periodontitis tissues (n=3) at D28. Scale bars=50 μm or 200μm. (D). Quantitative analysis of (C). *p < 0.05. (E). The draining cervical lymph nodes (left) and the spleen (right) of IgG and Anti-VISTA treated groups at D21 and D28.

**Table 1.1 The blood biochemistry of mice liver function at D28 (n=5)**

| GROUP | NO. | INDEX | RESULT | INDEX | RESULT |
| --- | --- | --- | --- | --- | --- |
| IgG | 1 | ALT | 64.330 | AST | 95.635 |
|  | 2 |  | 60.018 |  | 83.148 |
|  | 3 |  | 58.749 |  | 93.459 |
|  | 4 |  | 74.452 |  | 104.970 |
|  | 5 |  | 74.121 |  | 125.483 |
| Anti-VISTA | 6 | ALT | 65.493 | AST | 87.693 |
|  | 7 |  | 85.396 |  | 139.545 |
|  | 8 |  | 61.109 |  | 79.284 |
|  | 9 |  | 96.841 |  | 115.554 |
|  | 10 |  | 83.030 |  | 108.545 |
| Reference ranges | | 10.06-96.47(U/L) | | 36.31-235.48(U/L) | |

**Table 1.2 The blood biochemistry of mice renal function at D28 (n=5)**

| GROUP | NO. | INDEX | RESULT | INDEX | RESULT |
| --- | --- | --- | --- | --- | --- |
| IgG | 1 | BUN | 29.881 | CR | 25.877 |
|  | 2 |  | 29.403 |  | 23.904 |
|  | 3 |  | 29.515 |  | 8.553↓ |
|  | 4 |  | 27.361 |  | 15.351 |
|  | 5 |  | 29.219 |  | 16.228 |
| Anti-VISTA | 6 | BUN | 29.818 | CR | 14.693 |
|  | 7 |  | 23.742 |  | 23.026 |
|  | 8 |  | 27.075 |  | 17.544 |
|  | 9 |  | 34.089 |  | 11.842 |
|  | 10 |  | 29.349 |  | 14.035 |
| Reference ranges | | 10.81-34.74 (mg/dL) | | 10.91-85.09 (μmol/L) | |

**Table 2.1 The blood biochemistry of mice liver function at D21 (n=4)**

| GROUP | NO. | INDEX | RESULT | INDEX | RESULT |
| --- | --- | --- | --- | --- | --- |
| IgG | 1 | ALT | 89.14 | AST | 130.00 |
|  | 2 |  | 79.91 |  | 102.78 |
|  | 3 |  | 93.03 |  | 134.01 |
|  | 4 |  | 92.13 |  | 115.41 |
| Anti-VISTA | 6 | ALT | 91.29 | AST | 124.94 |
|  | 7 |  | 79.95 |  | 119.03 |
|  | 8 |  | 98.32↑ |  | 160.08 |
|  | 9 |  | 94.35 |  | 145.08 |
| Reference ranges | | 10.06-96.47(U/L) | | 36.31-235.48(U/L) | |

**Table 2.2 The blood biochemistry of mice renal function at D21 (n=4)**

| GROUP | NO. | INDEX | RESULT | INDEX | RESULT |
| --- | --- | --- | --- | --- | --- |
| IgG | 1 | BUN | 30.53 | CR | 28.72 |
|  | 2 |  | 33.14 |  | 44.54 |
|  | 3 |  | 29.41 |  | 22.89 |
|  | 4 |  | 33.59 |  | 44.54 |
| Anti-VISTA | 6 | BUN | 29.59 | CR | 13.74 |
|  | 7 |  | 34.30 |  | 37.88 |
|  | 8 |  | 27.12 |  | 42.46 |
|  | 9 |  | 25.41 |  | 25.81 |
| Reference ranges | | 10.81-34.74 (mg/dL) | | 10.91-85.09 (μmol/L) | |
